# The effect of music training on students’ mathematics and physics development at middle schools in China: A longitudinal study

**DOI:** 10.1016/j.heliyon.2024.e27702

**Published:** 2024-03-09

**Authors:** Yanan Zhou, Jian Lian

**Affiliations:** a School of Art, Beijing Foreign Studies University, Beijing, China; b Shandong Management University, Jinan, China

**Keywords:** Mathematics, Physics, Music training, ANCOVA, Middle school

## Abstract

As a descriptive-inferential study, this research aimed at revealing the relationship between music training and academic development with the Chinese middle school students' academic performance of mathematics and physics skills. The participants of this study consisted of the students from two different middle schools located at two cities in Shandong province, China. From each school 250 students were selected, and the statistics was used to analyze both the academic performance of the students and the data obtained from the scale designed by the authors. The research results show that the non-music students outperformed music students on both mathematics and physics development. In addition, music training did not contribute to the academic achievement independently but rather integrated with several factors like parents’ education and out-of-school engagement. The findings suggest the positive influence on non-musical cognitive learning, and it has potential implications for the Chinese middle school education.

## 1 Introduction

The Chinese University entrance examination plays a significant role in the national livelihood in China. Due to the huge change happening in the requirement of Chinese University entrance examination, an increasing number of students in China chooses to take an art examination as a main substitution or at least an approach of adding bonus points in the entrance examination. As a mainstream subject in the art examination, music education has attracted great attention from the students and teachers. Thus, the need to investigate the relationship between music training and academic achievement specifically in China has been more prominent than ever. Previously, a spate of research has been proposed to unveil the correlation between music and cognitive benefits [[1], [2], [3]]. The work of [4] pointed that the music education may stimulate the activities of brain and thus could positively affect mathematics performance. However, the influence of music training on academic achievement at Chinese middle school level still remains unresolved.

Past research has shown the universal impact of music may relate to the academic achievement. However, most of the previous research also showed that music was only one of the important factors that could affect mathematics performance, e.g., the parents’ education. Additionally, most studies on the academic performance of music instruction were based on the cases in developed countries not the data collected from China.

### 1.1 State of the literature

• Music training could enhance the stimulus like brain activities, which is conceived as the main source of relationship between music and mathematics.
• The research on the influence of music training on academic achievement mainly focuses on the short-term investigation.
• There are many challenges regarding the influence of music education on academic performance. The specific situation in China is a challenge as the rapid development of education in China.

### 1.2 Contribution of this paper to the literature

• Music training did significantly correlate with the mathematics achievement during the middle school students' development.
• Meanwhile, music training did significantly correlate with the physics achievement during the middle school students' development.
• This study found that there was no significant group-wise difference between the students from Jinan and Qingdao. The results were consistent between the two school districts of different location and socioeconomic status.

The present study investigates the relationship between music training and academic performance of students at the middle school in China. A sample of 250 students from several middle schools in Jinan that is the provincial capital of Shandong and 250 students from the other middle schools in Qingdao that is also an important city in Shandong, China were selected. The comparison of school districts are shown in Table 1.

**Table 1:** The comparison of school districts Jinan and Qingdao in 2015.

Item	Jinan	Qingdao
Setting	Urban city	Urban city
Population	7.91 million	4.88 million
Median income	63,996￥	76,116￥

The academic records including the examination grades (mathematics and physics) and the data collected through surveys were analyzed statistically to produce necessary evaluations.

### 1.3 Literature review

A great deal of educators and parents always believed that out-of-school music training or music instruction could negatively affect the academic achievements and grades of the students. Some of the educators even suggested removing the music classes from the school to enhance the students’ academic performance. The study of [5] expressed that there was no significant difference in sixth-grade reading, language and mathematics achievement among the students who excused from regular classroom activities for the study of instrumental music and the students not studying instrumental music. It would seem that this is a negative relationship between academic achievement and music education. However, it also demonstrated that the executed academic period had no effect on the musical students. Meanwhile, four districts that differ in size, setting, socioeconomic level, and racial composition were included in this study, and the comparison statistics results were consistent among four different districts.

The work of [6] conducted a study on comparing the mathematical achievement and overall academic of students with music credits and students without music credits at a high school in USA. The results showed that there was no statistically significant difference in their mean mathematics score and the mean cumulative grade point averages (GPAs), and a slight upward trend in GPAs existed along with the increase of music credit. In this study, only one high school was included. Thus, there was no correlation analysis between different schools from a variety of districts. Although the results found by Refs. [5,6] both revealed there was no significant difference between the musical students and non-musical students, they both admitted that music there is a link between mathematics and music. The work of [4] reviewed the empirical evidence relative to the influence of music on the intellectual, social and personal development of children and youngsters. This study explained the transfer effect of musical skills on other activities. The work of [2] also pointed that the students with good temporal-spatial reasoning ability could display promising music achievement, and the people with good musical performance often show good mathematical intelligence.

A descriptive study on 13,000 students that compared music students and non-music students in their academic grades was conducted [7]. The results demonstrated that music students outperformed the non-music students in mathematics, language and history, while the largest difference was found in English. 30 undergraduates were tested with music and no music, the group comparison results showed that the students who were listening to the music did better than the students were not listening to the music during the test [8]. Besides the above-mentioned research, a plethora of studies have revealed the similar findings. But they did not show that music students were smarter than non-music students. In addition, the work of [13], the brain activity during high and low working memory load conditions of musical transposing versus math calculations in classically trained musicians was compared to Magnetoencephalography (MEG), which was used to examine musical cognition and the neural consequences of music training. In 2023, Akin [14] conducted a fixed-effects meta-analysis to unpack the causal role of music interventions on mathematics achievement. The findings in this study indicated that music interventions had a individually small to moderate positive effect on mathematics achievement.

### 1.4 Research questions

This study aimed at assessing the relationship between music training and academic achievements of the musical students and non-musical students at middle schools from two different districts in China. To avoid the influence of different districts on the academic performance, we first investigated the district consistency of the two cities mentioned above. Following were the research questions of the study.

1. Were the results consistent between two school districts that differ in setting, population and income?
2. Whether music training was relative to the improvement of middle school students' mathematics development?
3. Whether music training was relative to the improvement of middle school students' physics development?
4. Whether music training independently contributes to the students' academic achievement?

## 2 Research Methodology

### 2.1 Ethics statement

This study was approved by the Ethics Committee of Ocean University of China, with ethics approval reference 2,022,071,106. The INFORMED CONSENT was obtained from all participants and the Parents for our study.

The present study was a descriptive-inferential study combined with the survey research. In addition, the parametric contrast statistics was exploited since the data collected in this study follows a normal distribution and many studies in the literature [[6], [7], [8]] adopted the same strategy as our study.

## 3 Participants and materials

The population of the study was the students of middle schools in Jinan and Qingdao districts. A sample of 250 students from (135 females) three middle schools in Jinan that is the provincial capital of Shandong and 250 students (129 females) from three middle schools in Qingdao that is the other city in Shandong were randomly selected. All of the students entered their middle schools at September in 2010, which is also taken as the date of enrollment by the other middle schools at Jinan and Qingdao in 2010. At that time, the mean age of the students was 13.4 years old (SD = 0.6). To be notable, the training experience of the students was collected through questionaires, in which following questions need to be answered, including: (1) whether the students have received formal music training; (2) whether the students have obtained regular music training; (3) the frequency of their own music training; (4) the duration of their average music training class. The students who have taken at least one category of music instruction like vocal training or instrumental training for more than 6 months were labeled as music students, while the other students are marked as non-music students, the corresponding statistical information are listed in Table 2. The music students had amean duration of training of 31.23 months (SD = 6.94), a monthly frequency of training with 6.8 times (SD = 0.71) and a monthly duration of practice for 7.3 h (SD = 0.65) based on the survey carried out in the students’ final semester of their 2016–2017 academic year.

**Table 2:** The number of students, the statistics of parents' education, and the out-of-school engagement data at Jinan and Qingdao.

District	Group	n	Parents' Education time (year)		Out-of-school engagement time duration per week (hours)	
			Mean	SD	Mean	SD
**Jinan**	Music	113	14.6	0.82	4.2	0.47
	Non-music	137	14.9	0.91	4.6	0.53
**Qingdao**	Music	122	15.1	0.77	3.9	0.37
	Non-music	128	14.8	0.69	4.3	0.29

The music students' and non-music students’ mathematics and physics development data from semester 1 to 12 were included in this study. Both the students from Jinan and Qingdao were asked to complete the same standardized tests on mathematics and physics to eliminate the difference of examination paper. The tests and the marking criterion were both supplied by an educational testing service authorized by Shandong Provincial Government. Five teachers jointly graded the examination papers, and major voting was used when divergence appeared. The range of score was from 0 to 150 for mathematics and physics tests.

Besides the academic records, parents' education and out-of-school academic engagement were also taken into consideration in the present study. On the one hand, the parents were required to complete a survey on their educational background in the final semester, in which the years of education, the final academic degree and the parents' majors were collected (partially shown in Table 2) as [9] reported that the education of mother was a sensitive indicator of students' academic achievement. Meanwhile, we combined the parents' education into our study to assess if the music training independently contribute to the students’ academic performance.

On the other hand, the students were asked to provide the out-of-school engagement time duration of academic activities (music training was not included) through a survey carried out in the final semester of the students, and the corresponding information was demonstrated in Table 2.

In this study, the reliability Cronbach's alpha coefficient of the rating scale was 0.94, while the Cronbach's alpha coefficients of the sub-scales of engagement, attention, and achievement were 0.88, 0.89, and 0.86, which indicated promising reliability of the measures. In addition, we implemented the validation factor analysis for the scale using Amos software, for all the 25 questions with RMSEA<0.05, factor loadings >0.7, X^2^/df < 2, and CFI>0.9, and there was significant correlation between the dimensions (p < 0.05). This demonstrates the good validity of the measures.

### 3.1 Data analysis

The data collected though test scores and surveys was analyzed by the authors using SPSS 22.0. There are three main analysis in this study.

1. The ANOVA compared the students in Jinan and Qingdao to assess whether there was a significant difference between the two groups.
2. To identify the relationship between the music and academic achievement of the middle school students in China, two multilevel models were used for evaluations including average, variance of performance on mathematics and physics at each semester at the middle schools in Jinan and Qingdao. The multilevel models can accurately capture the interactions between variables at different levels, such as individuals and classes. The 12 semesters and the two groups (music and non-music) served as repeated measure and the between-subject variable, respectively. To test the autogressive (AR) effect of the scores of mathematics and physics from 12 different semesters, the AR(1) model was initially exploited. With the outcome generated, no autoaggresive effect had been detected. This AR test contributed to evaluate the correlation between students' grades during different semesters of their study, and ensured that the assessment of the impact of music training was not affected by previous grades.
3. The bi-variate correlation analysis was used to assess the correlations between music training, parents' education, out-of-school academic engagement and students' academic performance. Regression analysis was performed on the independent contribution of music training, parents' education and out-of-school engagement to reveal if music training independently contribute to the academic performance.

## 4 Results

### 4.1 The consistency between the two middle school districts

**Fig. 1:**
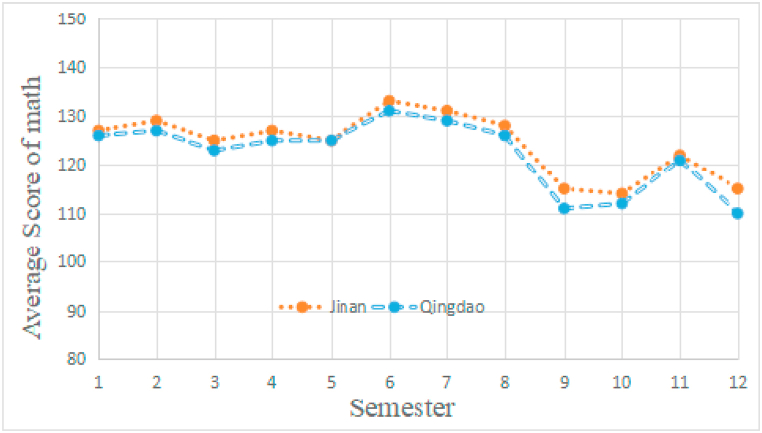
The students' mathematics development in Jinan and Qingdao. Independent *t*-test unveiled that the students in Jinan marginally outperformed students in Qingdao at all of the semesters (p < 0.05). There was no significant difference between the two school districts.

**Fig. 2:**
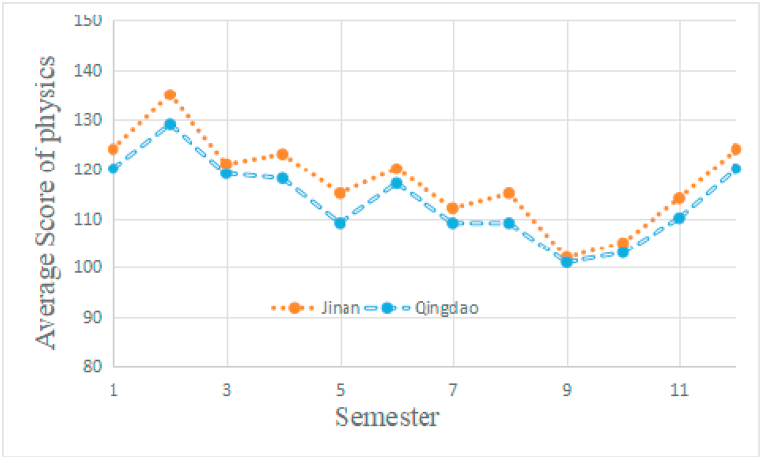
The students' physics development in Jinan and Qingdao. Independent *t*-test unveiled that the students in Jinan marginally outperformed students in Qingdao at all of the semesters (p < 0.05). There was no significant difference between the two school districts.

Two multilevel models analyzed the students’ achievement on mathematics, physics at two cities, respectively. Both the analysis revealed significant there were no significant difference between the two school districts (Fig. 1, Fig. 2). Then, separate ANOVA compared the students in Jinan and Qingdao to assess whether there was a significant difference in academic achievement between the two groups of students.

**Fig. 3:**
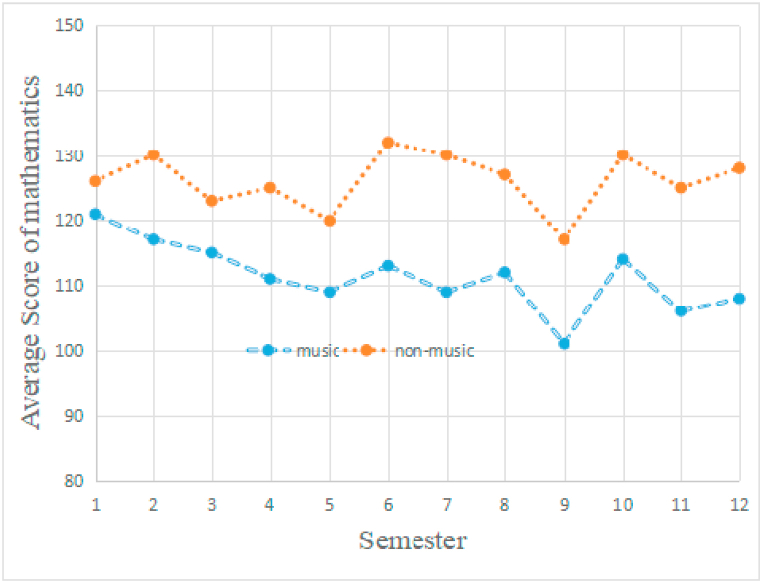
The students' mathematics development. Independent *t*-test unveiled that the non-music students outperformed music students at semesters 2, 3, 4, 5, 6, 7, 8, 9, 10, 11 and 12 (p < 0.05). And there was a significant difference between music and non-music students at semester 1.

**Fig. 4:**
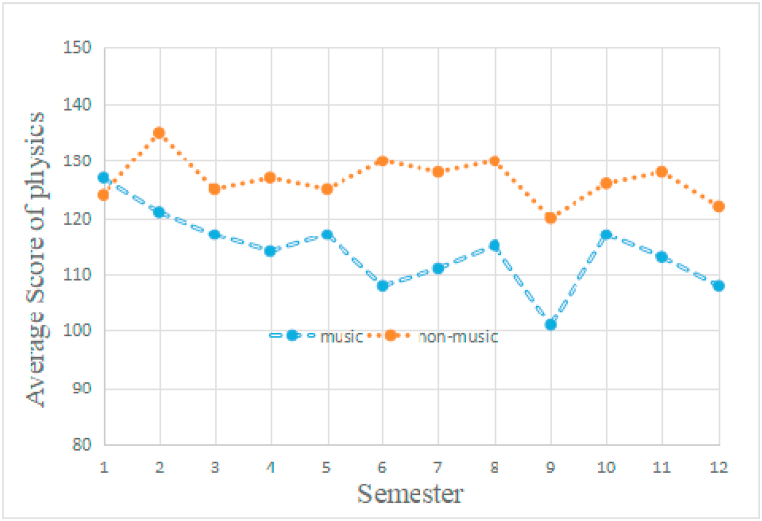
The students' physics development. Independent *t*-test unveiled that the non-music students outperformed music students at semesters 2, 3, 4, 5, 6, 7, 8, 9, 10, 11 and 12 (p < 0.05). The music students' physics score was marginally better than non-music students at semester 1.

There was no significant difference between the two school districts (shown in Fig. 3, Fig. 4), the which demonstrated that the academic achievement between Jinan and Qingdao were consistent with each other.

### 4.2 The influence of music training on the students’ academic achievement

Two multilevel models analyzed the students’ achievement on mathematics, physics, respectively. Both the analysis revealed a significant interaction of time-group (Z = 2.36, p < 0.05), suggesting there were significant difference between music and non-music students on mathematics and physics performance (Figs. 3 and 4).

As shown in Fig. 3, the non-music students' performance on mathematics was better at almost all of the semesters except for semester 1 (p < 0.05) than music students'. Fig. 4 showed the similar phenomenon, the non-music students’ achievement on physics was also better than music students except for semester 1(p < 0.05). And the music training was relative to detrimental of academic achievement in this study.

### 4.3 The independent contribution of music training to students’ academic performance

By using bi-variate correlation analysis, the correlations between music training, parents' education, out-of-school academic engagement and students' academic performance was assessed. Then, the regression analysis was executed to reveal the independence of music training, parents’ education and out-of-school engagement to the academic achievement, respectively.

The results revealed that the students' mathematics and physics performance related to music training (r = 0.18, p < 0.05), parents' education (r = 0.22, p < 0.05) and out-of-school academic engagement (r = 0.29, p < 0.05). By using regression analysis, we found that music training and out-of-school academic engagement independently contributed to the students’ academic achievement.

## 5 Discussion

We first excluded the influence of the academic tests difference (both the students from Jinan and Qingdao took the same test papers) and the district difference (the results shown by Fig. 1, Fig. 2 showed there is no significant different between the two cities). The following assessment suggested: first, there was a significant difference between the music students and non-music students; second, music training not only affected but also independently influenced the academic achievement of the middle school students in this study. To note that the work [6] found no statistically significant difference in the mathematics performance and music credit. The results of [7] proved that the music students outperformed the non-music students in mathematics. Meanwhile, the study of [8] observed that the students listening to the music achieved better mathematics performance over the students that were not listening to the music [8]. Different from results obtained by the previous research [[10], [11], [12]], which revealed a positive correlation or no correlation between students’ participation in music and the corresponding academic achievement, the present study found there was negative correlation between music training and academic performance. This is probably because the time period occupied by music training has significantly affected the middle school students in China. Another reason may reside in the special learning environment nurtured by the rigorous Chinese University entrance examination. As long as the students chose to take the art examination as a substitution of regular examination, they would spend much more time on art related courses (e.g., music) and pay less attention on the academic courses like mathematics and physics. Notable that there is a distinct difference between the music students in previous research and the present study, most of the music students in our study did not take music as their hobbies but rather a key to the gate of University. Thus, the musical training of the students could significantly impair the academic performance. We proposed to decrease music training from curriculum design and designing customized music education projects for specific student groups.

## 6 Conclusion

The focus of this study was on music participation and academic achievement, but the degrees of each student achievement in musical activities had not been differentiated. This study also has several limitations. First, the sample size is small and did not allow generalization. Second, only mathematics and physics have been taken into consideration in the current study. Finally, we found that music training, parents’ education and out-of-school academic engagement independently contributed to the academic achievement, respectively. Thus, more relative factors such as learning motivation and environmental effect should be included. Future research should be carried to investigate how does the different achievement of the individual student in music affects the overall academic performance. For instance, does the level of music training have an influence on the academic achievement?

## Data availability statement

The data used in this study is available on request from the corresponding author.

## CRediT authorship contribution statement

**Yanan Zhou:** Writing – review & editing, Writing – original draft, Project administration, Investigation, Funding acquisition, Formal analysis, Conceptualization. **Jian Lian:** Writing – review & editing, Writing – original draft, Visualization, Validation, Software, Methodology, Investigation, Formal analysis.

## Declaration of competing interest

The authors declare that they have no known competing financial interests or personal relationships that could have appeared to influence the work reported in this paper.
